# The Detailed Analysis of Polish Patients with Non-Small Cell Lung Cancer Through Insights from Molecular Testing (POL-MOL Study)

**DOI:** 10.3390/ijms252111354

**Published:** 2024-10-22

**Authors:** Dariusz M. Kowalski, Magdalena Zaborowska-Szmit, Maciej Bryl, Agnieszka Byszek, Dariusz Adam Dziedzic, Piotr Jaśkiewicz, Renata Langfort, Maciej Krzakowski, Tadeusz Orłowski, Rodryg Ramlau, Sebastian Szmit

**Affiliations:** 1 Department of Lung Cancer and Thoracic Tumors, Maria Sklodowska-Curie National Research Institute of Oncology, 02-781 Warsaw, Poland; dariusz.kowalski@nio.gov.pl (D.M.K.); magdalena.zaborowska-szmit@nio.gov.pl (M.Z.-S.); piotr.jaskiewicz@nio.gov.pl (P.J.); maciej.krzakowski@nio.gov.pl (M.K.); 2 Polish Lung Cancer Study Group, 01-138 Warsaw, Polandagnieszka.byszek@polgrp.org.pl (A.B.); drdariuszdziedzic@gmail.com (D.A.D.); r.langfort@igichp.edu.pl (R.L.); t.orlowski@igichp.edu.pl (T.O.); rramlau@gmail.com (R.R.); 3 Centre of Pulmonology and Thoracic Surgery, 60-569 Poznań, Poland; 4 Department of Thoracic Surgery, National Institute of Tuberculosis and Pulmonary Disease, 01-138 Warsaw, Poland; 5 Department of Pathology, National Institute of Tuberculosis and Pulmonary Disease, 01-138 Warsaw, Poland; 6 Institute of Oncology, Poznan University of Medical Sciences, 60-514 Poznań, Poland; 7 Department of Cardio-Oncology, Centre of Postgraduate Medical Education, 01-813 Warsaw, Poland; 8 Department of Cancer Diagnostics and Cardio-Oncology, Maria Sklodowska-Curie National Research Institute of Oncology, 02-781 Warsaw, Poland

**Keywords:** molecular testing, non-small lung cancer, squamous cell carcinoma, genetic mutations

## Abstract

Molecular testing is recommended in patients with metastatic non-small cell lung cancer (NSCLC), but the extent of its use in Poland is unknown. The aim of the POL-MOL study was to investigate the frequency of using molecular testing in Polish patients with NSCLC. The invited Polish oncologists completed two questionnaires, and data for 1001 patients undergoing systemic treatment for NSCLC were collected. The use of molecular tests for the following genetic mutations was recorded: *EGFR* (del19, sub21), *EGFR* (other than del19/sub21), *EGFR* T790M, *ALK* (expression and rearrangement), *RET*, *NTRK*, *ROS1*, *BRAF*, *HER2*, and *MET*, as well as for immunochemical assessment of programmed cell death ligand 1 (PD-L1). Thanks to the weighting procedure, the results are representative of the population of Polish patients treated for NSCLC. Molecular tests were applied in 78% of patients with NSCL, 70% of patients with NSCLC not otherwise specified, and in 12% of patients with squamous cell carcinoma of the lung. The frequency of application increased with disease stage in all groups. In patients with squamous cell carcinoma, approximately 30% of tests for *EGFR*, *ALK*, and *RET* mutations were positive, which confirms the importance of testing at least a preselected subgroup of patients.

## 1. Introduction

Lung cancer is one of the most common cancers worldwide, with almost 2.5 million new cases diagnosed every year [1,2,3]. It is also the most common cause of death due to malignant tumors globally, accounting for 1.8 million deaths per year [1,2,3]. It is prognosed to become the leading cause of death by 2060, with an estimated 2.4 million deaths annually [4,5].

In Poland, lung cancer is the most frequent malignant cancer, with almost 30,000 new cases diagnosed in 2020, which accounts for 15% of all oncologic diagnoses [6,7,8]. It is also the main cause of cancer-related deaths and the cause of almost 6% of all deaths [6,8]. An increase in new cases is predicted in the next decades due to population aging, as the incidence of lung cancer increases considerably among patients aged 65 years or older. According to the World Health Organization (WHO), the incidence of malignant neoplasms in Poland is expected to increase by 20% by 2040 [2,8].

Most patients are diagnosed in an advanced stage of the disease. In Poland, the diagnosis is most often made in stage III (24–38% of cases) and stage IV (47–62% of cases), depending on the region [9,10]. This is because the disease remains asymptomatic for a long time, and diagnostic procedures are initiated only at a later stage [2]. This results in a high mortality-to-incidence ratio, with 5-year survival rates of approximately 10% to 15% [6,11,12]. 

Clinically, lung cancer is divided into small cell lung cancer (approx. 15% of cases) and NSCLC (approx. 85% of cases) [2,11,13,14,15]. Based on histology, NSCLC is further classified into adenocarcinoma (35–45% of all diagnosed primary lung cancers), squamous cell carcinoma (approx. 30%), large cell carcinoma (2–10%), non-small cell carcinoma not otherwise specified (NOS), and other rare morphological types [2,15,16,17,18,19]. Sometimes, NSCLC is classified into two broad types, squamous and non–squamous cell carcinoma [20]. 

Traditionally, NSCLC was treated using surgery, chemotherapy, and radiation therapy [21]. However, the study of molecular biology of NSCLC, and especially the discovery that driver mutations or other oncogene alterations are present in more than half of all adenocarcinomas, has revolutionized NSCLC diagnosis and treatment [22]. Major mutations found in NSCLC are as follows: Kirsten rat sarcoma viral oncogene homolog, *KRAS*, gene mutations (15–30%); epidermal growth factor receptor, *EGFR*, gene mutations (10–35%); anaplastic lymphoma kinase, *ALK*, and *ROS-1* gene rearrangement (3–7% and 1–3%, respectively); *BRAF* mutation (1–5%); neurotrophic tyrosine receptor kinase, *NTRK*, fusion (0.2–3%); *MET* mutation or amplification (2–4%); *RET* mutation or fusion (0.6–1%); and human epidermal growth factor receptor-2, *HER2*, mutation (2–5%) [1,16,20,23,24]. 

Specific mutations in *EGFR* have been shown to sensitize tumors to a targeted treatment with tyrosine kinase inhibitors [25,26,27,28]. In 2009, the first-line administration of EGFR tyrosine kinase inhibitors became standard [29]. In more recent years, several new drugs directly targeting various mutations have been approved, such as ALK inhibitors, ROS1 inhibitors, and BRAF inhibitors, recommended if tumors contain certain molecular alternations [30].

Concurrently, progress in understanding immunology and antitumor immune responses has led to the development of immunotherapy agents like programmed cell death 1 (PD-1) and programmed cell death ligand 1 (PD-L1) checkpoint inhibitors (anti-PD-1 and anti-PD-L1 antibodies) that improve the immune system’s capacity to recognize and delete tumors [1,30]. Molecular therapies and immunotherapy are currently recommended in advanced or metastatic NSCLC. Patients with a druggable molecular alteration who are cured with targeted therapy benefit from significantly higher response rates, longer progression-free survival, and improved quality of life [22,31]. The 5-year survival rate for metastatic NSCLC is approximately 6% for patients treated with chemotherapy and 15% to 50% for patients treated with targeted therapies or immunotherapies [32]. The move towards biomarker-based treatment approaches has made genetic or molecular testing a standard of care in advanced NSCLC [11,33,34]. Defining the molecular characteristics of neoplastic tissue by identifying specific mutations or gene expression rearrangements is crucial for tumor classification, predicting disease progression and prognosis, and choosing the optimal treatment [16,35]. There are two types of biomarkers used in the diagnosis of NSCLC: predictive and prognostic. Predictive biomarkers are those for which specific targeted therapies exist (e.g., *EGFR* or *ALK* mutations), while prognostic biomarkers are indicators of tumor aggressiveness and as such can help predict patient survival independent of the treatment received [32].

According to current recommendations, molecular testing should be performed immediately after the diagnosis of NSCLC and prior to the initiation of therapy. The results can then be used to guide initial therapy selection [24,30,36]. As genetic alterations in oncogenic drivers are most frequent in patients with adenocarcinoma, biomarker testing has been recommended in non-squamous NSCLC [36,37,38]. However, it has been suggested that young patients without adenocarcinoma histology and without a history of smoking may have a higher likelihood of alterations in oncogenic drivers, so molecular testing should be considered in these patients [37]. However, according to the most recent guidelines of the National Comprehensive Cancer Network (NCCN), routine molecular testing should also be considered in all patients with metastatic squamous NSCLC, because these patients also have actionable biomarkers, even though not as frequently as patients with non-squamous NSCLC [32].

The NCCN recommends that all NSCLC patients with advanced disease should be screened for the most common biomarkers for which approved targeted therapies or immunotherapy exist. These include *EGFR*, *ALK*, *ROS1*, and *BRAF* mutations, along with the immunochemical assessment of programmed cell death ligand 1 (PD-L1 expression level). Moreover, broader molecular testing is strongly advised, including less frequent mutations such as *MET*, *RET*, *HER2*, and *NTRK* [24,30,32,36].

As targeted therapy is applied mostly in metastatic disease, broad molecular testing is recommended in stage IV of NSCLC. Currently, the *EGFR* mutation is the only biomarker that should be tested in early NSCLC [32]. However, the introduction of comprehensive testing for stage I–III disease has recently been proposed. This approach can help guide the selection of adjuvant treatment, provide access to clinical trials, facilitate rapid treatment decisions in the event of recurrence, and aid in risk stratification for disease relapse. Tests proposed for earlier stages of NSCLC include all targetable genetic alterations with therapies approved for use in the advanced setting (i.e., *EGFR*, *ALK*, *ROS1*, *BRAF*, *NTRK1/2/3*, *MET* exon 14 skipping, and *RET*) as well as PD-L1, *HER2*, and *KRAS* [39].

Although broad molecular testing is recommended by current guidelines at least in advanced NSCLC, some reports suggest that it is used less frequently than it should be. Testing rates of less than 80% were reported for *EGFR* mutations, which were the first biomarkers established for NSCLC [39]. The rates can be even lower for stages I–III of the disease, given that not all guidelines recommend molecular testing in these earlier stages [39].

In Poland, molecular/biomarker testing is offered to patients with non-squamous NSCLC. It usually includes testing for *EGFR* mutation and *ALK/ROS1* rearrangement. Recently, some institutions introduced next-generation sequencing with other targetable alterations, such as *BRAF*, *MET*, *RET*, *HER2*, and *NTRK1-3*, in conjunction with dedicated clinical research programs. However, selective reimbursement of tests and an insufficient number of pathologists might limit the scale of testing [6]. There are no country-level data regarding the frequency of molecular testing in NSCLC patients. The only existing analyses report data from single regions and for a small subset of tests (*EGFR*, *ALK*, *ROS*, and PD-L1). In addition, no detailed data for each separate test are available [40,41]. Understanding the extent to which molecular testing is used in NSCLC patients in Poland, along with identifying barriers to personalized treatment, might be the first step to increase the use of molecular testing. The availability of targeted therapies for Polish patients could improve the quality of care and treatment outcomes [11,41]. Therefore, the aim of this study was to explore the extent of molecular testing in NSCLC patients in Poland. Given that some recommendations suggest that molecular tests should particularly be used in patients with non-squamous carcinoma, this study focused on subgroups of patients with NSCLC.

## 2. Results

### 2.1. Patient Characteristics

Overall, data for 1001 patients with NSCLC were collected. The mean age of patients was 63.9 years (median, 65 years; range, 28–85 years). Patients aged 55 to 65 years constituted 29% of patients, and those aged 66 years or older -37%. In terms of histological subtypes, 51% of patients were diagnosed with adenocarcinoma, 36% with squamous cell carcinoma, 3% with large cell carcinoma, 2% with adenosquamous carcinoma, and 8% with NOS. On initiation of pharmacological treatment, 7%, 33%, and 59% of patients were classified as stage II, stage III, and stage IV disease, respectively. Most patients (72%) were assessed as Eastern Cooperative Oncology Group grade 1. The characteristics of patients with squamous NSCLC, non-squamous NSCLC, and NOS did not differ significantly from those of the whole sample. Detailed characteristics of patients are presented in Table 1.

### 2.2. Molecular Testing

**Figure 1:**
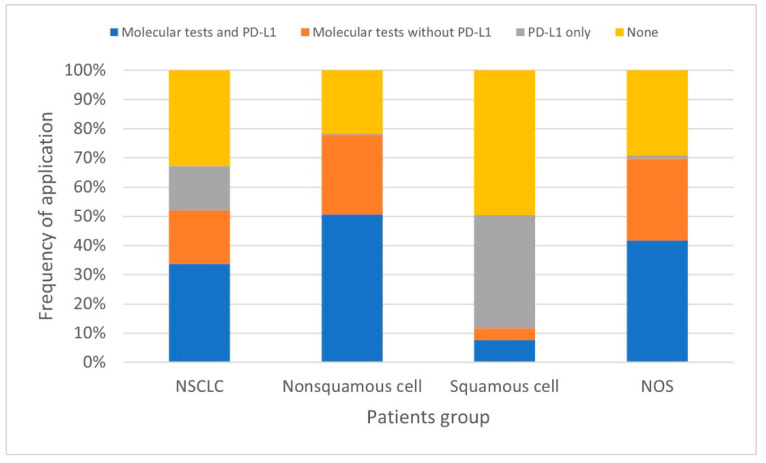
Frequency of molecular and PD-L1 testing in all NSCLC patients and patient subgroups classified by cancer type.

Of all NSCLC patients, 34% underwent both molecular testing and PD-L1 assessment, 18% underwent molecular testing only, and 15% underwent PD-L1 testing only. Molecular tests were not performed in 33% of the patients. Regarding cancer types, the pattern of testing was similar in patients with non-squamous NSCLC and NOS, while it differed significantly in patients with squamous NSCLC (Figure 1; Cramer’s V = 0.487, *p* < 0.001). 

Among patients with non-squamous NSCLC and NOS, 51% and 42%, respectively, underwent both molecular testing and PD-L1 assessment, as compared with 8% of patients with squamous NSCLC. Molecular testing without PD-L1 assessment was performed in 27% of patients with non-squamous NSCLC, 28% of patients with NOS patients, and only 4% of patients with squamous NSCLC. In contrast, PD-L1 assessment alone was performed in 39% of patients with squamous NSCLC and only in 1% of those with non-squamous NSCLC and 1% of those with NOS. Neither molecular testing nor PD-L1 assessment was performed in 22% of patients with non-squamous NSCLC, 29% of patients with NOS patients, and 49% of patients with squamous NSCLC (Table 2).

**Figure 2:**
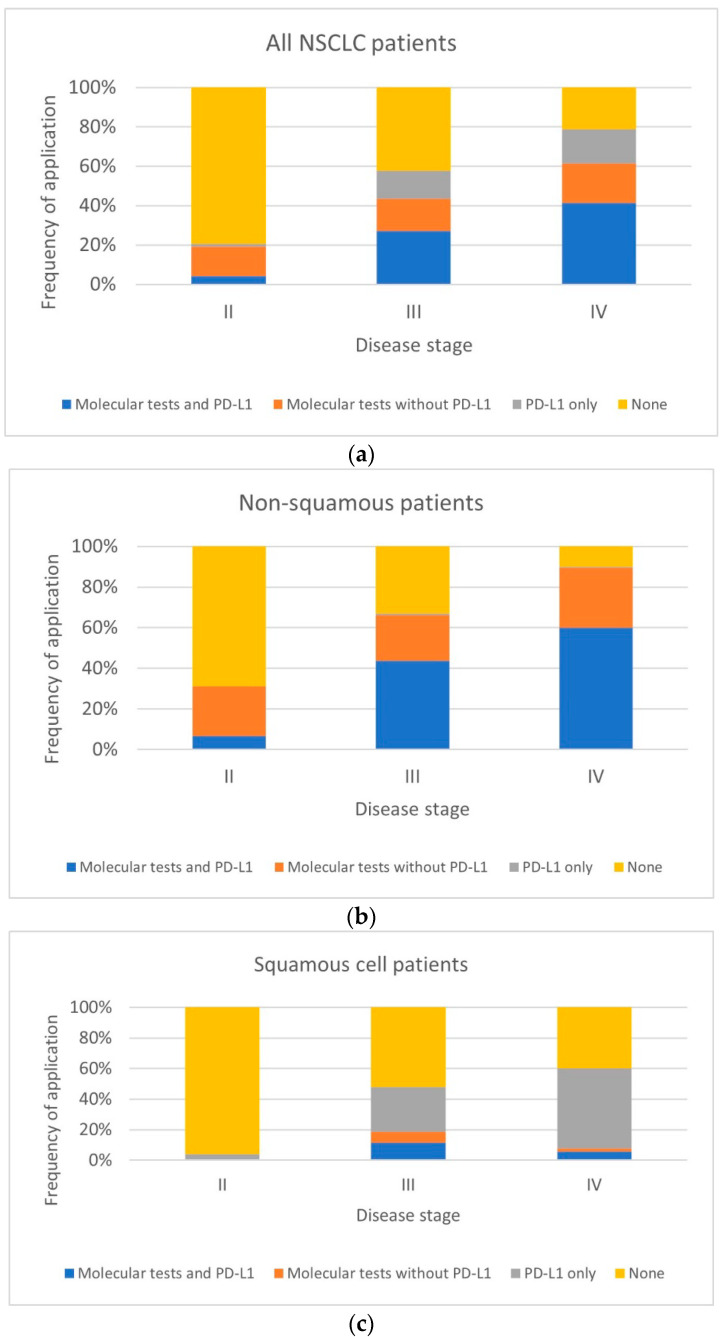

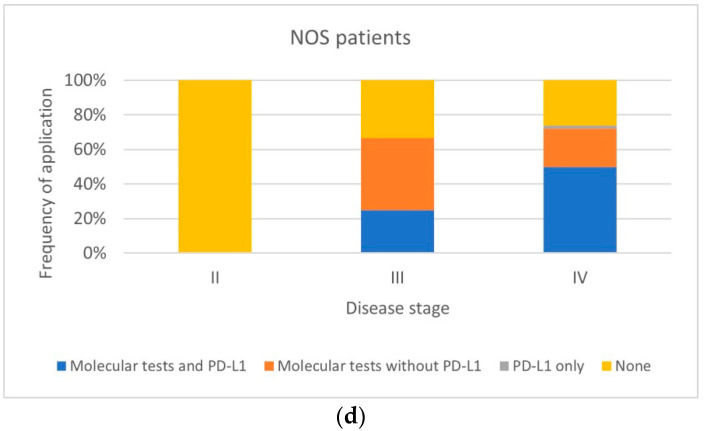
Frequency of molecular testing with disease stage in NSCLC patients and patient subgroups classified by cancer type: (**a**) All NSCLC patients, (**b**) Non-squamous patients, (**c**) Squamous cell patients, (**d**) NOS patients.

The frequency of using molecular tests increased with disease stage both in the whole study population and in separate subgroups (Figure 2).

In patients with non-squamous NSCLC, the frequency of molecular testing and PD-L1 assessment increased with disease stage, while the frequency of molecular tests without PD-L1 was stable. In patients with NOS, no tests were applied in those with stage II disease, while at stages III and IV, the proportion of molecular tests with PD-L1 increased and that of molecular tests without PD-L1 decreased, resulting in a similar overall proportion of molecular tests performed in these two stages. In patients with squamous NSCLC, the proportion of PD-L1 tests increased with disease stage, while the proportion of other tests, which was relatively low in stage III, decreased. Detailed results are presented in Table 3. 

The proportion of tests performed versus ordered was similar: only 49 patients had at least one test ordered that was not performed. In 21% of these cases, the reason was low-quality tissue; in another 21%, an insufficient amount of tissue; and in 12%, both low-quality tissue and an insufficient amount of tissue. In another 17% of cases, the decision not to perform additional tests was made based on positive *EGFR* test results. In the remaining 29% of cases, the reason was “other” or unknown. 

**Figure 3:**
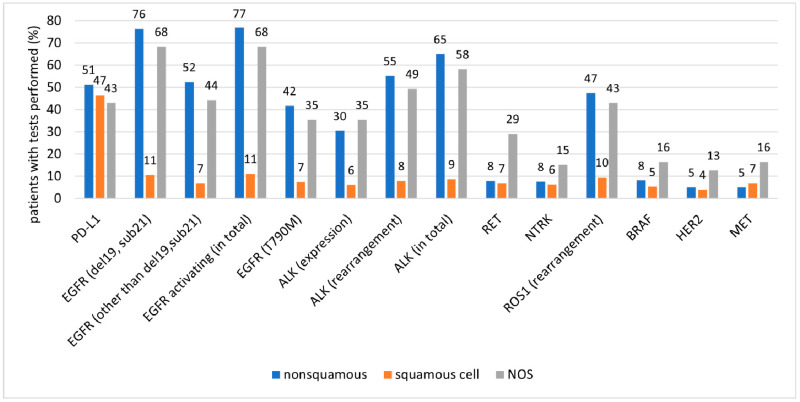
Proportion of performed tests in patient subgroups classified by cancer type.

**Figure 4:**
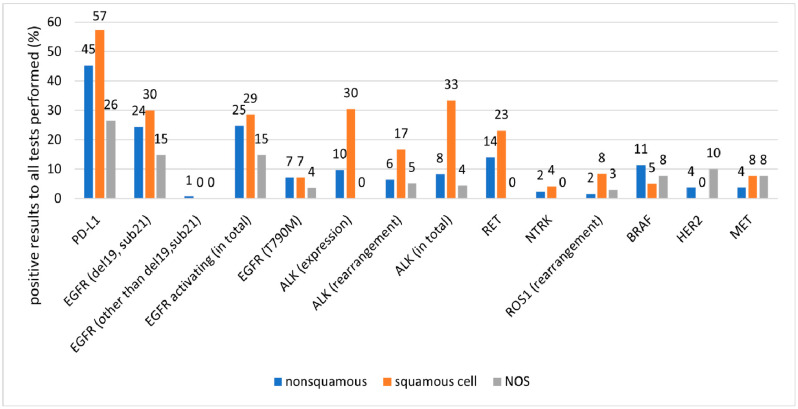
Proportion of positive results relative to the number of molecular tests performed in patient subgroups classified by cancer type.

**Figure 5:**
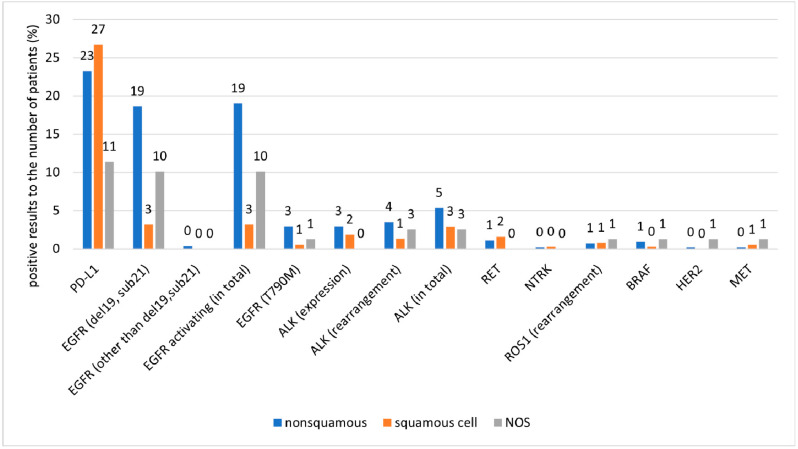
Proportion of positive results relative to the number of patients in patient subgroups classified by cancer type.

The proportion of the tests performed and of positive results by cancer type are presented in Figure 3, Figure 4 and Figure 5 and in Table 4. 

**Figure 6:**
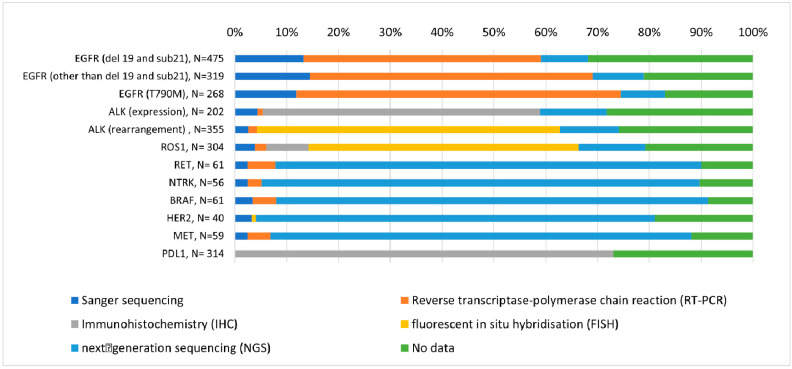
Methods used for molecular testing of patients with adenocarcinoma.

In patients with adenocarcinoma, *EGFR*, *ALK*, and *ROS1* mutations as well as PD-L1 expression were mostly tested using single biomarker assays, such as Sanger sequencing, reverse transcriptase-polymerase chain reaction, immunohistochemistry, and fluorescent in situ hybridization. In contrast, less common mutations (*RET*, *NTRK*, *BRAF*, *HER2*, and *MET*) were tested mostly in cases where next-generation sequencing (NGS) was used (Figure 6). Overall, NGS was used in 8% of patients with adenocarcinoma who underwent molecular testing.

## 3. Discussion

Our findings indicate that one-third of patients with NSCLC underwent both molecular and PD-L1 expression assays. In addition, 52% of patients underwent molecular testing either alone or in combination with PD-L1 assay. These proportions were higher in patients with non-squamous NSCLC and NOS (79% and 70%, respectively) than in those with squamous NSCLC. In advanced NSCLC, molecular tests were applied in 61% of all patients, 89% of those with non-squamous NSCLC, and 72% of those with NOS. While the higher frequency of using molecular tests in non-squamous NSCLC and NOS versus the entire group was observed for established mutations, the differences for emerging mutations such as *RET*, *NTRK*, *BRAF*, *HER2*, and *MET* were negligible (approx. 10% in both groups).

Direct comparison with other studies is difficult due to differences in the assays used and patient populations studied. In Poland, only two studies reported the frequency of molecular testing for established biomarkers in individual regions. In a study that assessed the prevalence of *EGFR* mutations in patients with non-squamous NSCLC in Lubelskie and Wielkopolskie voivodeships, the frequency of testing for these mutations was estimated to be approximately 40% in 2011–2012 [40]. In a more recent study, molecular tests (*EGFR* and/or *ALK* activating mutations, and/or *ROS1* rearrangements, and/or PD-L1 expression) were performed in 40% of patients with stage III lung cancer and 60% of those with stage IV lung cancer in the Dolnośląskie voivodeship in 2019–2020 [41]. 

Most studies from other countries reported testing rates for *EGFR* and *ALK* mutations, and the proportion of patients undergoing molecular diagnosis was relatively low, even though the use of biomarkers increased over time. A single-center study from the United States assessed the use of EGFR and ALK assays between 2010 and 2013 in patients with advanced non-squamous NSCLC. The proportion of patients tested was lower than 60%, although it increased each year from 37% to 58% for *EGFR* and from 4% to 40% for *ALK* [42]. In another American study, 59% of patients with advanced non-squamous NSCLC were tested for both *EGFR* and *ALK* mutations between 2013 and 2015, and only 8% were tested for all seven mutations recommended by the NCCN [43]. In Belgium, 52.7% of patients with stage IV non-squamous NSCLC were tested for *EGFR* mutation in 2011 [44]. In a study comparing molecular treatment patterns in advanced NSCLC across Italy, Germany, Spain, Australia, Brazil, Taiwan, Japan, and Korea between 2011 and 2014, the proportion of patients with at least one molecular test performed varied from 43% in Brazil to 85% in Taiwan. For patients with non-squamous NSCLC, the proportion varied between 54% and 91% [45]. In several Central and Eastern European countries, at least 65% of eligible tumors were tested for *EGFR* mutations in 2014 [11]. In Switzerland, the frequency of testing for *EGFR* and *ALK* mutations among patients with advanced non-squamous NSCLC increased from 32% in 2009 to 79% in 2014 [46]. In Sweden, the proportion of patients with advanced non-squamous NSCLC tested for *EGFR* mutations was 49% in 2011-2012 and increased to 84% in 2019 [47,48]. A recent study from Germany, which assessed patients with advanced NSCLC between 2016 and 2019, reported that 92.2% of patients with non-squamous tumors and 70.7% of those with squamous tumors were tested for any biomarker before the initiation of the first-line treatment. The overall testing rates for the whole study period for non-squamous patients were 72.5% for *EGFR*, 74.5% for *ALK*, 66.1% for *ROS1*, 53.0% for *BRAF*, and 64% for PD-L1. The testing rates increased over time, from 80.8% in 2015/2016 to 88.9% in 2019. A significant increase was observed for emerging biomarkers (i.e., *BRAF*, *MET*, *RET*, and *HER2*) among patients with non-squamous NSCLC [49]. An American study, which assessed data from 2016-2019 for newly diagnosed patients with stage IV NSCLC, reported the following testing rates for patients with non-squamous NSCLC: 86% for *EGFR*, 84% for *ALK*, 77% for *ROS1*, 75% for PD-L1, and 62% for *BRAF*. Patients with non-squamous histology were tested less frequently, with a rate of 63% for PD-L1, 52% for *EGFR*, 50% for *ALK*, 46% for *ROS1*, and 35% for *BRAF*. The testing rates increased over time: 24% of patients with non-squamous NSCLC were tested for all five biomarkers in 2016 versus 70% in 2018; for squamous NSCLC, the respective values were 5% and 29% [50].

Our study showed relatively high testing rates for established biomarkers (especially *EGFR* and *ALK*) in patients with advanced non-squamous NSCLC and NOS, which is in line with other studies. However, in contrast to other studies, the testing rates for less common mutations were much lower in our population. Moreover, the testing rates were lower for patients with squamous NSCLC. Meanwhile, the most recent guidelines recommend that all patients with advanced NSCLC—both non-squamous and squamous—should be screened not only for *EGFR*, *ALK*, and PD-L1 but also for *ROS1* and *BRAF* mutations. Broader molecular testing for rare mutations (such as *MET*, *RET*, *HER2*, and NTRK) is strongly recommended in advanced disease, while in earlier stages, at least EGFR testing should be performed [24,32,36]. However, our results suggest that these recommendations are not followed in clinical practice in Poland. This is consistent with a recent report by the London School of Economics and Political Science, which found a significant gap between the demand for and provision of testing for some cancers, such as lung cancer, in Poland, as compared with other European countries [51]. It is important to note that in Poland, adjuvant therapy with osimertinib has been available for patients with the *EGFR* T790M mutation since 2017 and for patients with other *EGFR* mutations (del19, sub21) only since 2023 [52]. In our study, we collected data for patients diagnosed in 2019. Moreover, in recent years, three new substances were covered by drug programs in Poland: osimertinib, atezolizumab, and nivolumab. These changes will likely lead to an increase in the frequency of molecular testing among NSCLC patients. Further research is thus needed to assess the use of molecular testing after the introduction of these new therapies. 

Several studies investigated the reasons why biomarker testing is not performed in NSCLC patients. One of the most important reasons was the low quality of tissue samples and an insufficient amount of tissue. Other reasons were long turnaround time that could lead to treatment delays, lack of knowledge (including lack of awareness of guidelines, need for assistance in selecting appropriate tests for patients, lack of knowledge about NGS, poor understanding of molecular test reports), frailty precluding biopsy, and death shortly after diagnosis [48,49,53,54]. Another major reason was cost—either lack of reimbursement or regulations that prevented rapid ordering of tests [48,54]. Reimbursement has been identified as a key determinant of testing availability in Europe, with differences between Central and Eastern Europe and Western Europe. Limited reimbursement of tests and therapies in Central and Eastern Europe is an important barrier to molecular testing [48].

In Poland, targeted therapies and immunotherapies available for NSCLC patients and covered by the national insurance system include *EGFR*, *ALK*, *ROS1*, and PD-L1 inhibitors. The therapies are offered as part of a drug program to patients with specific cancer types confirmed by histological or cytological tests. The diagnostic standard is aligned with the availability of reimbursed molecular treatment, and testing is mostly offered to patients with non-squamous disease [6,35]. The lack of approved and reimbursed therapies for patients with rare mutations may explain the low testing rates for these mutations observed in our study. 

While a low proportion of patients with squamous NSCLC were tested for any mutation, a relatively high proportion of positive test results were observed in this group. This pattern can be explained by clinical practice in Poland. According to guidelines, patients without adenocarcinoma histology should be tested if there is a high probability of alterations in oncogenic drivers, i.e., in young patients without smoking history [37]. Our results suggest that screening based on these criteria is effective, allowing for the identification of patients who should receive targeted therapies. This demonstrates the importance of testing at least selected patients with squamous histology, as recommended by the guidelines. In our study, differences between the number of tests ordered and the number performed were negligible. In the rare cases where tests were ordered but not performed, inadequate tissue sample accounted for the lack of testing in 41% of cases. However, low testing rates were mostly related to low ordering rates due to lack of reasonable medical need. 

There are several ways to increase biomarker testing rates. First, education is important, as it can address the insufficient knowledge of recommendations and advanced testing methods, as well as inability to choose the right test and understand the results. Training professionals who collect tissue samples can help ensure that samples are of sufficient size and quality for molecular testing. Physician education can also help maximize the use of tissue and reduce the costs associated with repeated tissue biopsies [11,30,48,53]. Multidisciplinary teams consisting of clinicians, molecular pathologists, clinical molecular biologists, geneticists, and bioinformaticians can improve adherence to guidelines, including the use of gene-guided care, to ensure optimal diagnosis and treatment [48,53]. Finally, a real-world database could help address some of the problems with interpreting the results [48].

An important issue affecting the range of molecular tests performed is the use of single biomarker assays versus NGS. Initially, molecular testing used sequential single-gene testing, where changes in each gene were examined in a separate assay. This approach assumed that most oncogenic driver mutations were mutually exclusive, and it was efficient when only a few genes had to be screened. It was relatively cheap, easy to implement, and had a quick turnaround time. However, the number of molecular biomarkers has increased considerably in recent years, making single-gene testing ineffective because it would require additional testing or repeated biopsy to cover a wide range of genes [20,55,56,57]. Therefore, multiplexed assays that can assess multiple biomarkers in a single test, especially NGS, are becoming the standard [11,24]. NGS allows for simultaneous screening for both common and rare mutations that are less likely to be found with a single-gene approach [20,55,56,57]. It has been shown to be highly concordant with and more sensitive than traditional molecular tests [56,58]. NGS minimizes the use of tumor tissue, so it is useful when the amount of tissue is limited [48,59]. In addition, since NGS can detect a larger number of alterations, more patients can be offered targeted therapy, resulting in more years of life gained [48,57,58]. NGS also reduces the time to start appropriate targeted therapy because it eliminates the need for repeat testing and biopsies [57]. 

It was demonstrated that NGS is more cost-effective than single-gene testing when biomarkers other than *EGFR*, *ALK*, and *ROS1* are evaluated [56]. Additionally, in our study, rare mutations were mostly tested by NGS, which confirms the assumption that NGS is the most effective approach when several mutations need to be tested. However, the overall rate of NGS use was very low, reaching only 11% of cases. This proportion is considerably lower than that reported by other authors: 38.7% of non-squamous and 14.4% of squamous tumors in Germany or one-third of advanced NSCLC cases in the United States [49,50]. The low rate is most likely related to the fact that only a few tests and therapies are reimbursed in Poland. Therefore, it may still be more cost-effective to use a single-gene approach. It was suggested that the choice between single-gene testing and NGS is guided by costs, reimbursement rules, and approved treatments in a given country, as well as the specific needs of a patient, turnaround time, and the amount of tissue available [39]. In countries where reimbursement is limited to some biomarkers only, sequential performance of molecular testing can be used. The protocols take into account the order of frequency of the molecular alterations, starting with the most frequent. In all cases where the most frequent mutation is found, no further testing is required, as driver mutations are exclusive. This approach can increase the hit rate for different mutations and reduce the volume and cost of analysis by approximately 30% [23].

Limited reimbursement favors on-demand testing, in which molecular testing is ordered by the treating physician, over reflex testing, in which testing for specific, agreed-upon biomarkers is automatically ordered by the pathologist as soon as the NSCLC diagnosis is made [30,39,48]. Reflex testing can facilitate optimal use of tissue, increase testing rates, reduce the time to optimal treatment initiation due to early availability of results, and improve the quality of biomarker testing [11,20,30,39,48]. With reflex testing, higher mutation detection rates were observed, which is explained by the lack of preselection of patients based on clinical characteristics and the more comprehensive panel screened [39]. Other advantages include the ability to select patients for prospective clinical trials and retrospective patient outcome studies regarding both the prognostic value of different biomarkers and the impact of targeted treatment [39]. Reflex testing is the standard of care in other solid tumors, such as breast cancer [30]. 

The presented country-level data confirm that the frequency of molecular testing in NSCLC patients in Poland strongly depends on the type of cancer. For most common biomarkers for which reimbursement is offered, our results showed relatively high application rates in advanced non-squamous NSCLC and NOS and lower rates in squamous NSCLC. The high proportion of positive results in squamous NSCLC demonstrates the importance of testing at least a preselected subgroup of patients with a high probability of alterations in oncogenic drivers. The rates of testing for rare mutations were much lower, possibly due to the lack of reimbursed therapies. Single-gene testing was used in most cases where a small number of tests were performed. NGS was used to test the wider range of alterations in a small group of patients. 

Several targeted therapies were not available to Polish NSCLC patients at the time of data collection, and therefore the alterations targeted by these therapies were largely not addressed in this study. Therefore, limited reimbursement seems to be the main barrier to molecular testing in Poland.

## 4. Materials and Methods

The POL-MOL study was conducted by PEX PharmaSequence on behalf of the Polish Lung Cancer Group. Data were collected between September and December 2021. Clinicians described their NSCLC patients admitted between 1 July and 31 December 2019 and undergoing systemic treatment for NSCLC. The time period preceding the COVID-19 pandemic was deliberately chosen to avoid the effects of pandemic-induced changes in the functioning of the healthcare system.

Centers invited to participate in the study were chosen based on the drug programs for NSCLC patients in 2020. Centers with contracts below one million PLN, which together represented 10% of the total contract value in Poland, were excluded. The remaining 51 centers represented high, medium, and low potential as measured by the value of their contracts (Table 5). Centers from these three groups were included based on the contract size (i.e., the biggest centers in each group were invited first). No other criteria of center selection were applied. 

Twenty-one centers providing NSCLC treatment under drug reimbursement programs in 2019 agreed to participate in the study. Overall, they received 50% of the national contract value in this area and represented high, medium, and low potential as measured by contract value. A total of 21 physicians providing systemic therapy to NSCLC patients at these centers (one physician per center) were invited to complete two questionnaires: a basic and an extended version (Table 6). 

Inclusion criteria for patients were as follows: NSCLC diagnosis; systemic treatment for NSCLC in a participating center; admission between 1 July and 31 December 2019; and age of 18 years or older.

For each center, there was a maximum number of patients that could be included, depending on the contract size of the center (Table 1). This limitation aimed to reflect the structure of the centers in terms of contract size in the collected sample. 

The sample size for this study was determined based on a balance between methodological requirements, aimed at minimizing statistical error at a given confidence level, and the feasibility of funding the study. The incidence of lung cancer in Poland in 2018 was estimated at 22,000 cases, of which 85% were classified as NSCLC. It was assumed that 60% of NSCLC patients are diagnosed at a stage qualifying for systemic treatment (stage IIIb/IV). Based on these assumptions, the annual population of new NSCLC patients eligible for systemic treatment was estimated to be approximately 11,000. Therefore, a sample of 1001 patients was chosen to ensure data collection with a statistical error below 3% at a 95% confidence interval. A total of 1001 patients were described in the study, including 587 patients (58.64%) with adenocarcinoma or NOS for whom detailed data were collected.

Qualification for molecular testing necessary for optimal treatment of lung cancer patients was carried out in Poland based on available therapies within the “Drug Program B.6” of the Ministry of Health. At the stage of qualification of patients presented in the current study, the “Drug Program B.6” of July 2019 was in force, and the recommended molecular tests were as follows:

1. EGFR (possible use of afatinib, erlotinib, gefitinib as first line and osimertinib as second line in patients with mut T790 M) and criteria met:
  - Presence of an activating mutation in the EGFR gene encoding the epidermal growth factor receptor (EGFR) confirmed using a validated test performed in a laboratory with a current European quality control program certificate for the given test;
  - Presence of the T790M mutation in the EGFR gene confirmed using a validated test performed in a laboratory with a current European quality control program certificate for the given test;
2. ALK (crizotinib, alectinib) and ROS1 (crizotinib)—presence of rearrangement in the ALK gene based on immunohistochemistry (IHC) or fluorescence in situ hybridization (FISH) or new-generation sequencing (NGS) using a validated test performed in a laboratory with a current European quality control program certificate for the given test or presence of rearrangement in the ROS-1 gene based on fluorescence in situ hybridization (FISH) or new-generation sequencing (NGS) using a validated test performed in a laboratory with a current European quality control program certificate for the given test:
3. Immunotherapy with pembrolizumab as the first line in monotherapy in patients with high PD-L1 expression;
  - Presence of PDL1 expression in 50% or more of the tumor cells confirmed using the method indicated in the Product Characteristics or using DAKO 22C3 antibody concentrate or Ventana SP263 antibody;
  - Exclusion of EGFR gene mutations and ALK gene rearrangement in the case of adenocarcinoma, large cell or non-small cell lung cancer NOS using a validated test performed in a laboratory with a current European quality control program certificate for the given test;

The characteristics of molecular testing in Poland may be summarized as the following:

- NGS
  Next-generation sequencing (NGS) was used to identify mutations and gene fusions using the FusionPlex CTL Kit for Illumina, ArcherDx. The sequencing was performed using the MiniSeq (Illumina) instrument. The results were analyzed using Archer Analysis 5.1 and Archer Analysis 5.0 software. The scope of the analysis includes possible fusion variants of the following genes: ALK, ROS1, NTRK1/2/3, FGFR1/2/3, MET, NRG1, RET, and BRAF, and in the case of point mutations and deletions, insertions in the genes: ALK (T1151ins, L1152R, C1156Y, F1174L, L1196M, G1202R, S1206Y, 1269A); AKT (E17K); BRAF (G466V, G469, Y472, L597V, V600, D594G); DDR2 (S768R, T765P, G774); EGFR (variants in exons 18, 19, 20 and 21); HRAS (codons 12, 13 and 61); KRAS (codons 12, 13, 61, and 146); MAP2K1 (Q56P, K57N, D67N); MET (aberrant splice variant); NRAS (codons 12, 13, and 61); PIK3CA (E542K, E545, H1047); and ROS1 (G2032R). The average number of sequencing reads of the FusionPlex libraries was above 1,000,000 per sample. For DNA/RNA analysis, the average depth of coverage of the sequenced gene regions was not less than 500 reads; analytical sensitivity was 4% mutant DNA relative to normal DNA. The mutation detection rate was approximately 99.9% for mutations in the EGFR, KRAS, and BRAF genes in non-small cell lung cancer. For RNA analysis, the detection limit was not less than five fusion copies; analytical specificity was 99% for all known and new rearrangements of the ALK, RET, ROS1, NTRK1/2/3, FGFR1/2/3 genes. The indicated parameters are obtained when the neoplastic tissue constitutes no less than 20% in the preparation.
- qPCR
  DNA was isolated using the Agencourt FormaPure kit from Beckman Coulter. DNA concentration was assessed using the Quantus fluorimeter from Promega. EGFR gene status was assessed using the qPCR method using the commercial AmoyDx^®^ EGFR 29 Mutations Detection Kit. Analyzed mutations: p.Gly719Cys/Ser/Ala, deletions in exon 19, insertions in exon 20, p.Thr790Met, p.Ser768Ile, p.Leu858Arg, p.Leu861Gln. Mutations were named according to the HGVS nomenclature. Reference sequence number: (EGFR:LRG_304). Mutation detection rate: approximately 99% of EGFR mutations occurring in lung cancer.

During the study, data were collected on molecular testing for the following gene variant mutations: *EGFR* (del19, sub21), *EGFR* (other than del19/sub21), *EGFR* T790M, *ALK* (expression and rearrangement), *RET*, *NTRK*, *ROS1*, *BRAF*, *HER2*, and *MET*, as well as for immunochemical assessment of PD-L1. Data were weighted based on the contract size to reflect the entire population of NSCLC patients receiving systemic treatment in centers participating in drug programs in Poland. Therefore, the results presented are representative of the population of Polish patients treated for NSCLC. Kramer’s non-parametric V test was used in the data analysis.

## Figures and Tables

**Table 1:** Patient characteristics.

Characteristic	All Patients (*n* = 1001)		Non-Squamous NSCLC (*n* = 542)		Squamous NSCLC (*n* = 378)		NOS (*n* = 79)	
	Unweighted Data, % (*n*)	Weighted Data, %	Unweighted Data, % (*n*)	Weighted Data, %	Unweighted Data, % (*n*)	Weighted Data, %	Unweighted Data, % (*n*)	Weighted Data, %
Cancer Type								
Adenocarcinoma	51 (508)	48	94 (508)					
Large cell carcinoma	3 (34)	4	6 (34)					
NOS	8 (79)	9					100 (79)	
Squamouscell carcinoma	36 (356)	37			94 (356)			
Adenosquamous carcinoma	2 (22)	2			6 (22)			
No data	<1 (2)	0						
Sex								
Male	61 (615)	62	56 (304)	55	68 (257)	70	66 (52)	66
Female	39 (386)	38	44 (238)	45	32 (121)	30	34 (27)	34
Age (years)								
18–54	12 (119)	11	15 (84)	15	7 (28)	8	9 (7)	8
55–65	29 (287)	30	28 (151)	30	29 (108)	29	33 (26)	38
66–75	30 (300)	32	29 (158)	31	31 (118)	35	30 (24)	29
76+	7 (71)	8	6 (34)	7	7 (28)	9	11 (9)	16
No data	22 (224)	18	21 (115)	18	25 (96)	20	16 (13)	9
NSCLC stage at initiation of pharmacological treatment								
IIA	2 (17)	2	2 (10)	2	2 (7)	2	<1 (1)	0
IIB	6 (56)	4	6 (35)	5	5 (19)	4	1 (1)	0
IIIA	15 (150)	16	12 (65)	12	20 (76)	21	10 (8)	13
IIIB	18 (182)	18	16 (86)	16	21 (80)	20	20 (16)	22
IV	59 (588)	59	63 (343)	63	51 (191)	51	68 (54)	64
No data	1 (8)	0	1 (3)	0	1 (5)	1	0	0
ECOG Performance Status Scale grade at initiation of pharmacological treatment								
0	8 (77)	9	9 (48)	10	7 (27)	10	3 (2)	2
1	72 (722)	71	76 (412)	76	70 (264)	67	56 (44)	54
2	17 (170)	16	12 (65)	11	21 (78)	19	34 (27)	33
3	2 (19)	2	2 (11)	2	2 (7)	3	1 (1)	2
4	<1 (5)	1	<1 (1)	0	0	0	5 (4)	6
No data	1 (8)	1	1 (5)	1	1 (2)	1	1 (1)	3

**Table 2:** Frequency of using molecular testing and PD-L1 assessment. For two patients, the subtype of NSCLC is missing.

Tests Performed	All Patients (*n* = 1001)		Non-Squamous NSCLC (*n* = 542)		Squamous NSCLC (*n* = 378)		NOS (*n* = 79)	
	Unweighted Data, % (*n*)	Weighted Data, %	Unweighted Data, % (*n*)	Weighted Data, %	Unweighted Data, % (*n*)	Weighted Data, %	Unweighted Data, % (*n*)	Weighted Data, %
Molecular tests and PD-L1	34 (337)	33	51 (275)	53	8 (29)	6	42 (33)	37
Molecular tests without PD-L1	18 (184)	18	27 (147)	27	4 (15)	3	28 (22)	35
PD-L1 only	15 (151)	16	1 (3)	1	39 (147)	41	1 (1)	0
None	33 (329)	32	22 (117)	19	49 (187)	50	29 (23)	28

**Table 3:** Frequency of using molecular tests with disease stage in the entire population and subgroups by cancer type (Cramer’s V = 0.219, *p* < 0.001).

All Patients (*n* = 1001) *						
**Tests Performed**	**Stage II (*n* = 73)**		**Stage III (*n* = 332)**		**Stage IV (*n* = 588)**	
	**Unweighted Data, % (*n*)**	**Weighted Data, %**	**Unweighted Data, % (*n*)**	**Weighted Data, %**	**Unweighted Data, % (*n*)**	**Weighted Data, %**
Molecular tests and PD-L1	4 (3)	3	27 (90)	24	41 (244)	42
Molecular tests without PD-L1	15 (11)	25	17 (55)	18	20 (117)	18
PD-L1 only	1 (1)	1	14 (47)	16	18 (103)	19
None	79 (58)	71	42 (140)	42	21 (124)	22
**Non-squamous NSCLC (*n* = 542)**						
**Tests performed**	**Stage II (*n* = 45)**		**Stage III (*n* = 151)**		**Stage IV (*n* = 343)**	
	**Unweighted data, % (*n*)**	**Weighted data, %**	**Unweighted data, % (*n*)**	**Weighted data, %**	**Unweighted data, % (*n*)**	**Weighted data, %**
Molecular tests and PD-L1	7 (3)	6	44 (66)	41	60 (206)	64
Molecular tests without PD-L1	24 (11)	42	23 (34)	26	29 (101)	25
PD-L1 only	0	0	1 (1)	1	1 (2)	1
None	69 (31)	52	33 (50)	31	10 (34)	10
**Squamous NSCLC (*n* = 378)**						
**Tests performed**	**Stage II (*n* = 26)**		**Stage III (*n* = 156)**		**Stage IV**	
	**Unweighted data, % (*n*)**	**Weighted data, %**	**Unweighted data, % (*n*)**	**Weighted data, %**	**Unweighted data, % (*n*)**	**Weighted data, %**
Molecular tests and PD-L1	0	0	12 (18)	8	6 (11)	5
Molecular tests without PD-L1	0	0	7 (11)	5	2 (4)	1
PD-L1 only	4 (1)	1	29 (46)	33	52 (100)	54
None	96 (25)	99	52 (81)	55	40 (76)	40
**NOS patients (*n* = 79)**						
**Tests performed**	**Stage II (*n* = 1)**		**Stage III (*n* = 24)**		**Stage IV (*n* = 54)**	
	**Unweighted data, % (*n*)**	**Weighted data, %**	**Unweighted data, % (*n*)**	Weighted data, %	**Unweighted data, % (*n*)**	**Weighted data, %**
Molecular tests and PD-L1	0	0	25 (6)	26	50 (27)	43
Molecular tests without PD-L1	0	0	42 (10)	49	22 (12)	27
PD-L1 only	0	0	0	0	2 (1)	1
None	100 (1)	100	33 (8)	25	26 (14)	29

* No data for eight patients overall, including three non-squamous and five squamous cell patients.

**Table 4:** Number and proportion of tests performed and positive test results in all patients and subgroups by cancer type.

**All Patients (*n* = 1001)**						
**Test**	**Tests Performed**		**Positive Results Related to the Number of Patients**		**Positive Results Related to the Number of Tests Performed**	
	**Unweighted Data, % (*n*)**	**Weighted Data, %**	**Unweighted Data, % (*n*)**	**Weighted Data, %**	**Unweighted Data, % (*n*)**	**Weighted Data, %**
PD-L1	49 (488)	50	24 (236)	23	48 (236)	46
*EGFR* (del19, sub21)	51 (508)	50	12 (121)	11	24 (121)	21
*EGFR* (other than del19, sub21)	35 (345)	35	<1 (2)	0	1 (2)	0
*EGFR* activating (in total)	51 (513)	51	12 (123)	11	24 (123)	21
*EGFR* (T790M)	28 (282)	26	2 (19)	2	7 (19)	6
*ALK* (expression)	22 (216)	28	2 (23)	2	11 (23)	8
*ALK* (rearrangement)	37 (368)	38	3 (26)	2	7 (26)	6
*ALK* (in total)	43 (431)	44	4 (42)	3	10 (42)	8
*RET*	8 (82)	8	1 (12)	1	15 (12)	10
*NTRK*	8 (77)	8	<1 (2)	0	3 (2)	2
*ROS1* (rearrangement)	33 (327)	36	1 (8)	0	2 (8)	1
*BRAF*	8 (77)	8	1 (7)	0	9 (7)	6
*HER2*	5 (52)	5	<1 (2)	0	4 (2)	2
*MET*	8 (83)	8	<1 (5)	0	6 (5)	4
**Test**	**Tests performed**		**Positive results related to the number of patients**		**Positive results related to the number of tests performed**	
	**Unweighted data, % (*n*)**	**Weighted data, %**	**Unweighted data, % (*n*)**	**Weighted data, %**	**Unweighted data, % (*n*)**	**Weighted data, %**
PD-L1	51 (278)	54	23 (126)	21	45 (126)	40
*EGFR* (del19, sub21)	76 (414)	78	19 (101)	17	24 (101)	22
*EGFR* (other than del19, sub21)	52 (284)	56	<1 (2)	0	1 (2)	1
*EGFR* activating (in total)	77 (417)	79	19 (103)	17	25 (103)	22
*EGFR* (T790M)	42 (226)	40	3 (16)	3	7 (16)	6
*ALK* (expression)	30 (165)	43	3 (16)	3	10 (16)	8
*ALK* (rearrangement)	55 (299)	61	4 (19)	3	6 (19)	5
*ALK* (in total)	65 (352)	70	5 (29)	5	8 (29)	7
*RET*	8 (43)	9	1 (6)	1	14 (6)	8
*NTRK*	8 (41)	9	<1 (1)	0	2 (1)	1
*ROS1* (rearrangement)	47 (257)	56	1 (4)	0	2 (4)	1
*BRAF*	8 (44)	9	1 (5)	1	11 (5)	6
*HER2*	5 (27)	6	<1 (1)	0	4 (1)	2
*MET*	5 (27)	9	<1 (1)	0	4 (1)	3
**Test**	**Tests performed**		**Positive results related to the number of patients**		**Positive results to tests performed**	
	**Unweighted data, % (*n*)**	**Weighted data, %**	**Unweighted data, % (*n*)**	**Weighted data, %**	**Unweighted data, % (*n*)**	**Weighted data, %**
PD-L1	47 (176)	47	27 (101)	29	57 (101)	62
*EGFR* (del19, sub21)	11 (40)	8	3 (12)	2	30 (12)	26
*EGFR* (other than del19, sub21)	7 (26)	5	0	0	0	0
*EGFR* activating (in total)	11 (42)	8	3 (12)	2	29 (12)	25
*EGFR* (T790M)	7 (28)	5	1 (2)	0	7 (2)	7
*ALK* (expression)	6 (23)	4	2 (7)	1	30 (7)	27
*ALK* (rearrangement)	8 (30)	6	1 (5)	1	17 (5)	15
*ALK* (in total)	9 (33)	6	3 (11)	2	33 (11)	29
*RET*	7 (26	4	2 (6)	1	23 (6)	23
*NTRK*	6 (24)	4	<1 (1)	0	4 (1)	4
*ROS1* (rearrangement)	10 (36)	7	1 (3)	1	8 (3)	7
*BRAF*	5 (20)	3	<1 (1)	0	5 (1)	5
*HER2*	4 (15)	3	0	0	0	0
*MET*	7 (26)	4	1 (2)	0	8 (2)	8
**Test**	**Tests performed**		**Positive results related to the number of patients**		**Positive results related to the number of tests performed**	
	**Unweighted data, % (*n*)**	**Weighted data, %**	**Unweighted data, % (*n*)**	**Weighted data, %**	**Unweighted data, % (*n*)**	**Weighted data, %**
PD-L1	43 (34)	37	11 (9)	5	26 (9)	14
*EGFR* (del19, sub21)	68 (54)	71	10 (8)	10	15 (8)	15
*EGFR* (other than del19, sub21)	44 (35)	48	0	0	0	0
*EGFR* activating (in total)	68 (54)	71	10 (8)	10	15 (8)	15
*EGFR* (T790M)	35 (28)	37	1 (1)	1	4 (1)	2
*ALK* (expression)	35 (28)	41	0	0	0	0
*ALK* (rearrangement)	49 (39)	50	3 (2)	3	5 (2)	6
*ALK* (in total)	58 (46)	59	3 (2)	3	4 (2)	6
*RET*	29 (23)	17	0	0	0	0
*NTRK*	15 (12)	16	0	0	0	0
*ROS1* (rearrangement)	43 (34)	45	1 (1)	0	3 (1)	1
*BRAF*	16 (13)	17	1 (1)	1	8 (1)	4
*HER2*	13 (10)	13	1 (1)	1	10 (1)	5
*MET*	16 (13)	17	1 (1)	1	8 (1)	4

**Table 5:** Selection of participating centers.

Size of Drug Contract (PLN)	No. of Centers in Poland	% of the Total Contract Value in 2020	% of Contract Value after Exclusion of the Centers with <1 Million PLN Contracts	No. of Included Centers	Max. Number of Patients
>3 million	21	61	68	12	68
>1.5–3 million	20	23	25	7	36
>1–1.5 million	10	6	7	2	28
<1 million	42	10	-	-	-

**Table 6:** Data collection methods for the two questionnaires used in the study.

	Basic Questionnaire	Extended Questionnaire
Data collection	Retrospective	
Data collector	Physicians providing NSCLC treatment	
Data source	Medical records	
Population	Patients admitted to the center in the second half of 2019 and treated for NSCLC	Patients with adenocarcinoma or NOS
Aim	To collect data regarding the entire population of NSCLC patients receiving systemic treatment and to assess the frequency of molecular testing in this population	To collect more detailed data on molecular diagnostics and therapy in patients with adenocarcinoma or NOS
Topic/questions	Basic questions regarding NSCLC patients and molecular tests applied	Detailed questions regarding molecular diagnosis and treatment in patients with adenocarcinoma or NOS

## Data Availability

The data generated in the present study may be requested from the Polish Lung Cancer Group.

## References

[1] Padinharayil H., Varghese J., John M.C., Rajanikant G.K., Wilson C.M., Al-Yozbaki M., Renu K., Dewanjee S., Sanyal R., George A. (2022). Non-small cell lung carcinoma (NSCLC): Implications on molecular pathology and advances in early diagnostics and therapeutics. Genes Dis..

[2] Nalewaj K.P., Krawczyk P., Chmielewska I., Milanowski J. (2023). Delays in the diagnosis of lung cancer patients in Poland. Oncol. Clin. Pract..

[3] Bray F., Laversanne M., Sung H., Ferlay J., Siegel R.L., Soerjomataram I., Jemal A. (2024). Global cancer statistics 2022: GLOBOCAN estimates of incidence and mortality worldwide for 36 cancers in 185 countries. CA Cancer J. Clin..

[4] American Cancer Society (2018). Global Cancer Facts & Figures.

[5] Bray F., Ferlay J., Soerjomataram I., Siegel R.L., Torre L.A., Jemal A. (2018). Global cancer statistics 2018: GLOBOCAN estimates of incidence and mortality worldwide for 36 cancers in 185 countries. CA Cancer J. Clin..

[6] Adamek M., Biernat W., Chorostowska-Wynimko J., Didkowska J.A., Dziadziuszko K., Grodzki T., Jassem J., Kępka L., Kowalski D., Dziadziuszko R. (2020). Lung Cancer in Poland. J. Thorac. Oncol..

[7] European Union 2021 ECIS—European Cancer Information System. https://ecis.jrc.ec.europa.eu.

[8] Ferlay J., Laversanne M., Ervik M., Lam F., Colombet M., Mery L., Piñeros M., Znaor A., Soerjomataram I., Bray F. (2020). Global Cancer Observatory: Cancer Tomorrow.

[9] Didkowska J., Wojciechowska U., Śliwczyński A. (2019). Report on the Stages of Advancement, Treatment, and Survival of Lung Cancer Patients Registered with the National Cancer Registry (KRN) from 2014 to 2016.

[10] Wild C.P., Weiderpass E., Stewart B.W. (2020). World Cancer Report: Cancer Research for Cancer Prevention.

[11] Ryska A., Berzinec P., Brcic L., Cufer T., Dziadziuszko R., Gottfried M., Kovalszky I., Olszewski W., Oz B., Plank L. (2018). NSCLC molecular testing in Central and Eastern European countries. BMC Cancer.

[12] Yuan M., Huang L.L., Chen J.H., Wu J., Xu Q. (2019). The emerging treatment landscape of targeted therapy in non-small-cell lung cancer. Signal Transduct. Target. Ther..

[13] (2018). American Cancer Society: About Non-Small Cell Lung Cancer. https://www.cancer.org/content/dam/CRC/PDF/Public/8703.00.pdf.

[14] Howlader N., Forjaz G., Mooradian M.J., Meza R., Kong C.Y., Cronin K.A., Mariotto A.B., Lowy D.R., Feuer E.J. (2020). The effect of advances in lung-cancer treatment on population mortality. N. Engl. J. Med..

[15] Travis W.D., Brambilla E., Nicholson A.G., Yatabe Y., Austin J.H.M., Beasley M.B., Chirieac L.R., Dacic S., Duhig E., Flieder D.B. (2015). The 2015 World Health Organization Classification of Lung Tumors. J. Thorac. Oncol..

[16] Łaczmańska I., Dębicka I., Gil J., Michałowska D., Pawlak I., Sąsiadek M.M. (2021). Personalised medicine in lung cancer. Nowotw. J. Oncol..

[17] Kerr K.M., Bubendorf L., Edelman M.J., Marchetti A., Mok T., Novello S., O’Byrne K., Stahel R., Peters S., Felip E. (2014). Second ESMO consensus conference on lung cancer: Pathology and molecular biomarkers for non-small-cell lung cancer. Ann. Oncol..

[18] Travis W.D., Brambilla E., Noguchi M., Nicholson A.G., Geisinger K.R., Yatabe Y., Beer D.G., Powell C.A., Riely G.J., Van Schil P.E. (2011). International Association for the Study of Lung Cancer/American Thoracic Society/European Respiratory Society international multidisciplinary classification of lung Adenocarcinoma. J. Thorac. Oncol..

[19] Pikor L.A., Ramnarine V.R., Lam S., Lam W.L. (2013). Genetic alterations defining NSCLC subtypes and their therapeutic implications. Lung Cancer.

[20] Pujol N., Heeke S., Bontoux C., Boutros J., Ilié M., Hofman V., Marquette C.H., Hofman P., Benzaquen J. (2022). Molecular profiling in non-squamous non-small cell lung carcinoma: Towards a switch to next-generation sequencing reflex testing. J. Pers. Med..

[21] Popat S., Navani N., Kerr K.M., Smit E.F., Batchelor T.J.P., Van Schil P., Senan S., McDonald F. (2021). Navigating diagnostic and treatment decisions in non-small cell lung cancer: Expert commentary on the multidisciplinary team approach. Oncologist.

[22] Kris M.G., Johnson B.E., Berry L.D., Kwiatkowski D.J., Iafrate A.J., Wistuba I.I., Varella-Garcia M., Franklin W.A., Aronson S.L., Su P.F. (2014). Using multiplexed assays of oncogenic drivers in lung cancers to select targeted drugs. JAMA.

[23] Popper H.H., Tímár J., Ryska A., Olszewski W. (2014). Minimal requirements for the molecular testing of lung cancer. Transl. Lung Cancer Res..

[24] Aisner D.L., Riely G.J. (2021). Non–small cell lung cancer: Recommendations for biomarker testing and treatment. J. Natl. Compr. Cancer Netw..

[25] Besse B., Adjei A., Baas P., Meldgaard P., Nicolson M., Paz-Ares L., Reck M., Smit E.F., Syrigos K., Stahel R. (2014). 2nd ESMO consensus conference on lung cancer: Non-small-cell lung cancer first-line/second and further lines of treatment in advanced disease. Ann. Oncol..

[26] Smolle E., Pichler M. (2019). Non-smoking-associated lung cancer: A distinct entity in terms of tumor biology, patient characteristics and impact of hereditary cancer predisposition. Cancers.

[27] Travis W.D., Rekhtman N. (2011). Pathological diagnosis and classification of lung cancer in small biopsies and cytology: Strategic management of tissue for molecular testing. Semin. Respir. Crit. Care Med..

[28] Walsh S.L.F., Lederer D.J., Ryerson C.J., Kolb M., Maher T.M., Nusser R., Poletti V., Richeldi L., Vancheri C., Wells A.U. (2019). Diagnostic Likelihood Thresholds That Define a Working Diagnosis of Idiopathic Pulmonary Fibrosis. Am. J. Respir. Crit. Care Med..

[29] Mok T.S., Wu Y.L., Thongprasert S., Yang C.H., Chu D.T., Saijo N., Sunpaweravong P., Han B., Margono B., Fukuoka M. (2009). Gefitinib or carboplatin-paclitaxel in pulmonary adenocarcinoma. N. Engl. J. Med..

[30] Gregg J.P., Li T., Yoneda K.Y. (2019). Molecular testing strategies in non-small cell lung cancer: Optimizing the diagnostic journey. Transl. Lung Cancer Res..

[31] Hofman P., Rouleau E., Sabourin J.C., Denis M., Deleuze J.F., Barlesi F., Laurent-Puig P. (2020). Predictive molecular pathology in non-small cell lung cancer in France: The past, the present and the perspectives. Cancer Cytopathol..

[32] Ettinger D.S., Wood D.E., Aisner D.L., Akerley W., Bauman J.R., Bharat A., Bruno D.S., Chang J.Y., Chirieac L.R., Hughes M. (2021). NCCN Guidelines Insights: Non–Small Cell Lung Cancer, Version 2.2021: Featured Updates to the NCCN Guidelines. J. Natl. Compr. Cancer Netw..

[33] Sholl L.M., Aisner D.L., Varella-Garcia M., Berry L.D., Dias-Santagata D., Wistuba I.I., Chen H., Fujimoto J., Kugler K., Franklin W.A. (2015). LCMC Investigators. Multi-institutional Oncogenic Driver Mutation Analysis in Lung Adenocarcinoma: The Lung Cancer Mutation Consortium Experience. J. Thorac. Oncol..

[34] Dietel M., Bubendorf L., Dingemans A.M., Dooms C., Elmberger G., García R.C., Kerr K.M., Lim E., López-Ríos F., von Laffert M. (2016). Diagnostic procedures for non-small-cell lung cancer (NSCLC): Recommendations of the European Expert Group. Thorax.

[35] Pązik M., Mirowski M., Balcerczak E. (2022). Current Approach to Non-Small Cell Lung Cancer Diagnosis and Treatment—A Short Review.

[36] Kalemkerian G.P., Narula N., Kennedy E.B., Biermann W.A., Donington J., Leighl N.B., Lew M., Pantelas J., Ramalingam S.S., Sundaram B. (2018). Molecular Testing Guideline for the Selection of Patients with Lung Cancer for Treatment with Targeted Tyrosine Kinase Inhibitors: American Society of Clinical Oncology Endorsement of the College of American Pathologists/International Association for the Study of Lung Cancer/Association for Molecular Pathology Clinical Practice Guideline Update. J. Clin. Oncol..

[37] Lindeman N.I., Cagle P.T., Aisner D.L., Arcila M.E., Beasley M.B., Bernicker E.H., Colasacco C., Dacic S., Hirsch F.R., Yatabe Y. (2018). Updated molecular testing guideline for the selection of lung cancer patients for treatment with targeted tyrosine kinase inhibitors: Guideline from the College of American Pathologists, the International Association for the Study of Lung Cancer, and the Association for Molecular Pathology. Arch. Pathol. Lab. Med..

[38] Lindeman N.I., Cagle P.T., Beasley M.B., Chitale D.A., Dacic S., Giaccone G., Jenkins R.B., Kwiatkowski D.J., Saldivar J.S., Ladanyi M. (2013). Molecular testing guideline for selection of lung cancer patients for EGFR and ALK tyrosine kinase inhibitors: Guideline from the College of American Pathologists, International Association for the Study of Lung Cancer, and Association for Molecular Pathology. J. Thorac. Oncol..

[39] Aggarwal C., Bubendorf L., Cooper W.A., Illei P., Borralho Nunes P., Ong B.H., Tsao M.S., Yatabe Y., Kerr K.M. (2021). Molecular testing in stage I-III non-small cell lung cancer: Approaches and challenges. Lung Cancer.

[40] Krawczyk P., Ramlau R., Powrózek T., Wojas-Krawczyk K., Sura S., Jarosz B., Walczyna B., Pankowski J., Szumiło J., Milanowski J. (2012). The detection of EGFR mutations in patients with non-small cell lung cancer in selected molecular diagnostics centers in Poland. Kardiochir. Torakochirurgia Pol..

[41] Trembecki Ł., Sztuder A., Pawlak I., Matkowski R., Maciejczyk A. (2021). Analysis of lung cancer measures of the National Cancer Network pilot study in Poland for potential improvement in the quality of advanced-stage lung cancer therapy. BMC Cancer.

[42] Lim C., Tsao M.S., Le L.W., Shepherd F.A., Feld R., Burkes R.L., Liu G., Kamel-Reid S., Hwang D., Leighl N.B. (2015). Biomarker testing and time to treatment decision in patients with advanced nonsmall-cell lung cancer. Ann. Oncol..

[43] Gutierrez M.E., Choi K., Lanman R.B., Licitra E.J., Skrzypczak S.M., Pe Benito R., Wu T., Arunajadai S., Kaur S., Goldberg S.L. (2017). Genomic Profiling of Advanced Non-Small Cell Lung Cancer in Community Settings: Gaps and Opportunities. Clin. Lung Cancer.

[44] Vrijens F., De Gendt C., Verleye L., Robays J., Schillemans V., Camberlin C., Stordeur S., Dubois C., Van Eycken E., Van Meerbeeck J.P. (2018). Quality of care and variability in lung cancer management across Belgian hospitals: A population-based study using routinely available data. Int. J. Qual. Health Care.

[45] Lee D.H., Tsao M.S., Kambartel K.O., Isobe H., Huang M.S., Barrios C.H., Khattak A., de Marinis F., Kothari S., de Castro J. (2018). Molecular testing and treatment patterns for patients with advanced non-small cell lung cancer: PIvOTAL observational study. PLoS ONE.

[46] Ess S.M., Herrmann C., Frick H., Krapf M., Cerny T., Jochum W., Früh M. (2017). Epidermal growth factor receptor and anaplastic lymphoma kinase testing and mutation prevalence in patients with advanced non-small cell lung cancer in Switzerland: A comprehensive evaluation of real world practices. Eur. J. Cancer Care.

[47] Sandelin M., Berglund A., Sundström M., Micke P., Ekman S., Bergqvist M., Bergström S., Koyi H., Brandén E., Botling J. (2015). Patients with Non-small Cell Lung Cancer Analyzed for EGFR: Adherence to Guidelines, Prevalence and Outcome. Anticancer Res..

[48] Kerr K.M., Bibeau F., Thunnissen E., Botling J., Ryška A., Wolf J., Öhrling K., Burdon P., Malapelle U., Büttner R. (2021). The evolving landscape of biomarker testing for non-small cell lung cancer in Europe. Lung Cancer.

[49] Griesinger F., Eberhardt W., Nusch A., Reiser M., Zahn M.O., Maintz C., Bernhardt C., Losem C., Stenzinger A., Thomas M. (2021). CRISP Registry Group: Biomarker testing in non-small cell lung cancer in routine care: Analysis of the first 3717 patients in the German prospective, observational, nation-wide CRISP Registry (AIO-TRK-0315). Lung Cancer.

[50] Waterhouse D.M., Tseng W.Y., Espirito J.L., Robert N.J. (2021). Understanding Contemporary Molecular Biomarker Testing Rates and Trends for Metastatic NSCLC Among Community Oncologists. Clin. Lung Cancer.

[51] Gill J., Fontrier A.-M., Miracolo A., Kanavos P. (2020). Access to Personalised Oncology in Europe.

[52] Reimbursement Announcement of the Polish Ministry of Health no. 67 (1 January 2023). https://www.gov.pl/attachment/420adc35-6fb1-4060-8c7a-f215886f7a23.

[53] Mileham K.F., Schenkel C., Bruinooge S.S., Freeman-Daily J., Basu Roy U., Moore A., Smith R.A., Garrett-Mayer E., Rosenthal L., Silvestri G.A. (2022). Defining comprehensive biomarker-related testing and treatment practices for advanced non-small-cell lung cancer: Results of a survey of U.S. oncologists. Cancer Med..

[54] Association of Community Cancer Centers Operational Pathways for Biomarker Testing in NSCLC (2021). Environmental Scan. https://www.accc-cancer.org/docs/projects/operational-pathways-in-nsclc/accc-operational-pathways_final-(2).pdf.

[55] Naidoo J., Drilon A. (2014). Molecular Diagnostic Testing in Non-Small Cell Lung Cancer. Am. J. Hematol. Oncol..

[56] Guo H., Zhang J., Qin C., Yan H., Liu T., Hu H., Tang S., Tang S., Zhou H. (2022). Biomarker-Targeted Therapies in Non-Small Cell Lung Cancer: Current Status and Perspectives. Cells.

[57] Vanderpoel J., Stevens A.L., Emond B., Lafeuille M.H., Hilts A., Lefebvre P., Morrison L. (2022). Total cost of testing for genomic alterations associated with next-generation sequencing versus polymerase chain reaction testing strategies among patients with metastatic non-small cell lung cancer. J. Med. Econ..

[58] Zheng Y., Vioix H., Liu F.X., Singh B., Sharma S., Sharda D. (2022). Diagnostic and economic value of biomarker testing for targetable mutations in non-small-cell lung cancer: A literature review. Future Oncol..

[59] Isla D., Lozano M.D., Paz-Ares L., Salas C., de Castro J., Conde E., Felip E., Gómez-Román J., Garrido P., Enguita A.B. (2023). New update to the guidelines on testing predictive biomarkers in non-small-cell lung cancer: A National Consensus of the Spanish Society of Pathology and the Spanish Society of Medical Oncology. Clin. Transl. Oncol..

